# Cardiovascular disease, inflammation, and mRNA stability

**DOI:** 10.18632/aging.101619

**Published:** 2018-10-26

**Authors:** Allison B. Herman, Michael V. Autieri

**Affiliations:** 1 Department of Physiology, Independence Blue Cross Cardiovascular Research Center, Temple University School of Medicine, Philadelphia, PA 19140, USA

**Keywords:** mRNA stability, vascular smooth muscle cells, interleukin-19, FXR1

Despite nutritional modification and lipid reducing medications, atherosclerosis and other vascular inflammatory diseases continue to be a major medical and socioeconomic problem in our society, and will worsen as our population ages and becomes increasingly sedentary. Atherosclerosis is considered a lipid-driven inflammatory disease, and results from the recent CANTOS trial support the preeminent role of inflammation in this condition. Most studies focus on the role of pro-inflammatory cytokines in activation of macrophages, endothelial cells, and vascular smooth muscle cells (VSMC) in this disease. By comparison, a much smaller number of investigations focus on the role of endogenous counter-regulatory mechanisms in atherogenesis and their effects on these same cells.

The regulation of mRNA stability and translation are two levels of post-transcriptional regulation that permit resident vascular and immune cells to rapidly respond to inflammatory stimuli. Modification of mRNA stability proteins by anti-inflammatory factors in the vascular response to injury is an understudied topic, but could lead to therapeutic opportunities to treat vascular inflammatory diseases. Many labile mRNA transcripts, including pro-inflammatory cytokines, are tightly regulated due to cis-acting adenine and uridine-rich (AU-rich) elements (AREs) in their 3’ untranslated (UTR). Similar to cardiovascular disease, control of mRNA stability is dysregulated in aged tissue. One study described levels of RNA binding proteins (RBPs) differentially expressed in aged versus younger tissue, which has important implications for age-associated disease like atherosclerosis [1].

Interleukin-19 (IL-19) is an anti-inflammatory cytokine shown to reduce atherosclerosis in murine models [2]. Because IL-19 expression was higher in plaque compared with naïve artery, yet was capable of reducing atherosclerosis, it was concluded that IL-19 expression represented a compensatory, or counter-regulatory vascular response to injury. A recent study by Ray, et al. extended this report and crossed IL-19-/- mice with LDLR knockout mice to create double knock out (dKO) mice as a platform to test the impact of this interleukin in development of atherosclerosis [3]. As expected of a potent-anti-inflammatory cytokine, multiple modes of protection were identified by absence of IL-19 in this model, with the primary phenotype being exacerbation of atherosclerosis. Importantly, this phenotype could be rescued by i.p. injection of rmIL-19 into the dKO mice. Global effects on adaptive immunity included significantly increased expression of pro-inflammatory transcripts in spleen, as well as in bone marrow derived macrophages (BMDM) derived and cultured from dKO mice. Direct effects of lack of IL-19 on resident vascular cells (primarily VSMC) were manifested by significantly increased expression of pro-inflammatory transcripts in aortic arch and VSMC explanted and cultured from dKO. Further studies using the RNA polymerase inhibitor actinomycin D showed that the stability of inflammatory mRNA from BMDM and VSMC were significantly increased. Similar to in vivo experiments, this increased stability observed in dKO cells could be reversed by addition of IL-19. This complements an earlier study showing that addition of IL-19 to primary human VSMC decreased mRNA stability of several pro-inflammatory transcripts [4].

Human antigen R, (HuR) is a ubiquitously expressed member of the Hu (ELAV) family of RBPs, and is an important regulator of inflammatory mRNA stability. HuR is one of the best characterized RBPs and stabilizes numerous ARE-containing transcripts including those coding for cytokines, cell-cycle regulators and growth factors [5]. While a number of studies have implicated HuR as a pro-inflammatory mediator, there are no reports that suggest HuR activity or expression could be reduced by an anti-inflammatory modality. Several experiments identified an inverse relationship between IL-19 levels and HuR abundance. HuR protein, mRNA, and importantly, mRNA stability were increased in spleen, aortic arch, BMDM, and VSMC explanted from dKO mice. Again, this could be reversed by addition of rmIL-19 to cultured cells. HuR mRNA contains conserved and semi-conserved ARE in its 3’UTR, and is reported to regulate its own mRNA stability in a feedback mechanism [6]. Thus, one conclusion from this study is that IL-19 can decrease atherosclerosis by reduction in the mRNA stability of pro-inflammatory transcripts by reduction in HuR abundance.

**Figure 1:**
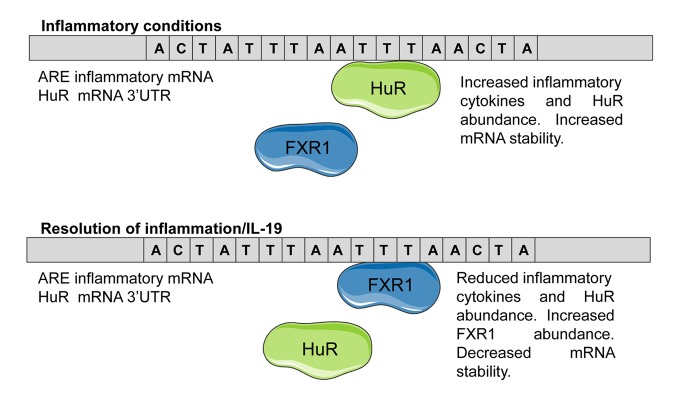
**Proposed working hypothesis of IL-19 reduction of inflammatory mRNA stability.** Under inflammatory conditions, HuR interacts with ARE in pro-inflammatory and HuR mRNA 3’UTR and stabilizes the transcript, resulting in increased inflammatory protein abundance. When inflammation is reduced, FXR1 expression increases, with the dual outcome of increased competition with HuR for ARE occupancy, as well as reduced HuR abundance. This results in reduced transcript stability.

In a search for a molecular mechanism for this effect, a recent study by Herman et al. identified and characterized an IL-19-inducible protein termed Fragile X-related protein (FXR1) [7]. FXR1 is an autosomal homologue of the FMR (Fragile X mental retardation) neuronal protein, and a presumed mRNA-binding protein. A muscle-enhanced protein, this study showed that FXR1 expression in VSMC was induced by IL-19 stimulation. In a proteomics-based interaction assay, FXR1 immunoprecipitated with HuR in primary human VSMC under pro-inflammatory, but not basal conditions. FXR1 bound to RNA, and ARE in particular. Importantly, the FXR1-HuR interaction was abolished by the addition of RNAse to the reaction mixture, suggesting that FXR1 and HuR were tethered by mRNA. The reduction in HuR protein abundance and mRNA stability observed when VSMC were stimulated with IL-19 was abolished when FXR1 was depleted by siRNA, suggesting that IL-19 anti-inflammatory effects, and particularly, reduction in HuR abundance, is mediated by FXR1. This implies a feed-forward mechanism whereby FXR1 not only displaces HuR on ARE of inflammatory transcripts, but also reduces HuR abundance itself, further changing the stoichiometry of ARE occupancy (Figure 1).

Modulation of mRNA stability has been posited as a promising therapeutic strategy, but there is little literature exploring the concept of RBPs as modulators of anti-inflammatory pathways and reduction of atherosclerosis. This work has the potential to identify mRNA stability proteins as a new class of targets for rational drug design.
